# The Seed Plant Flora of the Mount Jinggangshan Region, Southeastern China

**DOI:** 10.1371/journal.pone.0075834

**Published:** 2013-09-30

**Authors:** Lei Wang, Wenbo Liao, Chunquan Chen, Qiang Fan

**Affiliations:** 1 College of Resource Environment and Tourism, Capital Normal University, Haidian District, Beijing, P. R. China; 2 State Key Laboratory of Biocontrol and Guangdong Key Laboratory of Plant Resources, School of Life Sciences, Sun Yat-sen University, Guangzhou, Guangdong, P. R. China; 3 Jinggangshan Administration of Jiangxi Province, Jinggangshan, Jiangxi, P. R. China; Chinese Academy of Sciences, China

## Abstract

The Mount Jinggangshan region is located between Jiangxi and Hunan provinces in southeastern China in the central section of the Luoxiao Mountains. A detailed investigation of Mount Jinggangshan region shows that the seed plant flora comprises 2,958 species in 1,003 genera and 210 families (Engler’s system adjusted according to Zhengyi Wu’s concept). Among them, 23 species of gymnospermae belong to 17 genera and 9 families, and 2,935 species of angiosperms are in 986 genera and 201 families. Moreover, they can also be sorted into woody plants (350 genera and 1,295 species) and herbaceous plants (653 genera and 1,663 species). The dominant families are mainly Fagaceae, Lauraceae, Theaceae, Hamamelidaceae, Magnoliaceae, Ericaceae, Styracaceae, Aquifoliaceae, Elaeocarpaceae, Aceraceae, Rosaceae, Corylaceae, Daphniphyllaceae, Symplocaceae, Euphorbiaceae, Pinaceae, Taxodiaceae, Cupressaceae and Taxaceae. Ancient and relic taxa include *Ginkgo biloba*, *Fokienia* *hodginsii* , *Amentotaxus* *argotaenia* , *Disanthus* *cercidifolia* subsp. *longipes* , *Hamamelis* *mollis* , *Manglietia* *fordiana* , *Magnolia* *officinalis* , *Tsoongiodendron* *odorum* , *Fortunearia* *sinensis* , *Cyclocarya* *paliurus* , *Eucommia* *ulmoides* , *Sargentodoxa* *cuneata* , *Bretschneidera* *sinensis* , *Camptotheca* *acuminata* , *Tapiscia* *sinensis* , etc. The flora of Mount Jinggangshan region includes 79 cosmopolitan genera and 924 non-cosmopolitan genera, which are 7.88% and 92.12% of all genera. The latter includes 452 tropical genera (48.92%) and 472 temperate genera (51.08%). The temperate elements include 44 genera endemic to China, accounting for 4.76% of all genera. Among 1,003 genera, 465 have only a single species and 401 are oligotypic genera (with 2-5 species). These genera account for 86.34% of all genera. The floristic analysis indicates that the flora of Mount Jinggangshan region is closely related to the flora of Mount Wuyishan region in southeastern China. The flora of Mount Jinggangshan region also contains many elements of central and southern China. Mount Jinggangshan region is an important north-south floristic passageway and is also a boundary between the floras of eastern, central and south China.

## Introduction

The Mount Jinggangshan region is located in the center of the Luoxiao Mountains, an important, large, north-south trending mountain range in southeastern China. Compared with other larger mountain ranges, such as Mount Wuyishan, Nanling Mountains, Wuling Range, Mount Emeishan, Qinling Mountains, Hainan Mountains and Hengduan Mountains, the natural resources and biodiversity of the Luoxiao Mountains have been less well studied.

The Mount Jinggangshan region has four natural reserves: the Mount Jinggangshan National Nature Reserve, the Mount Qixiling National Nature Reserve and the Mount Nanfengmian National Nature Reserve, all of Jiangxi Province, and the Mount Taoyuandong National Natural Reserve of Hunan Province. During 1983-1984, the government of Jiangxi Province organized a comprehensive natural resource survey on the Mount Jinggangshan Nature Reserve [1]. Later, several institutions and scientists developed surveys and research projects on the biodiversity of the other three nature reserves [2-4]. Interestingly, the four reserves have the boundaries which are connected to each other, forming an integrated region. Based on a detailed research, it is stated that the flora of the Mount Jinggangshan region is the core of the Luoxiao Mountains’. Therefore, it is significant to comprehensively analyze the flora of the Mount Jinggangshan region.

Based on the field surveys, the specimens collected in the Mount Jinggangshan region from 2009 to 2012 and deposited in herbaria, and relevant literature, we compiled a checklist of the seed plant in the Mount Jinggangshan region [5]. Additionally, we analyzed the composition of the flora of the Mount Jinggangshan region in order to discuss its origin, evolution and floristic status.

## Geography

### Geographic Limits

**Figure 1:**
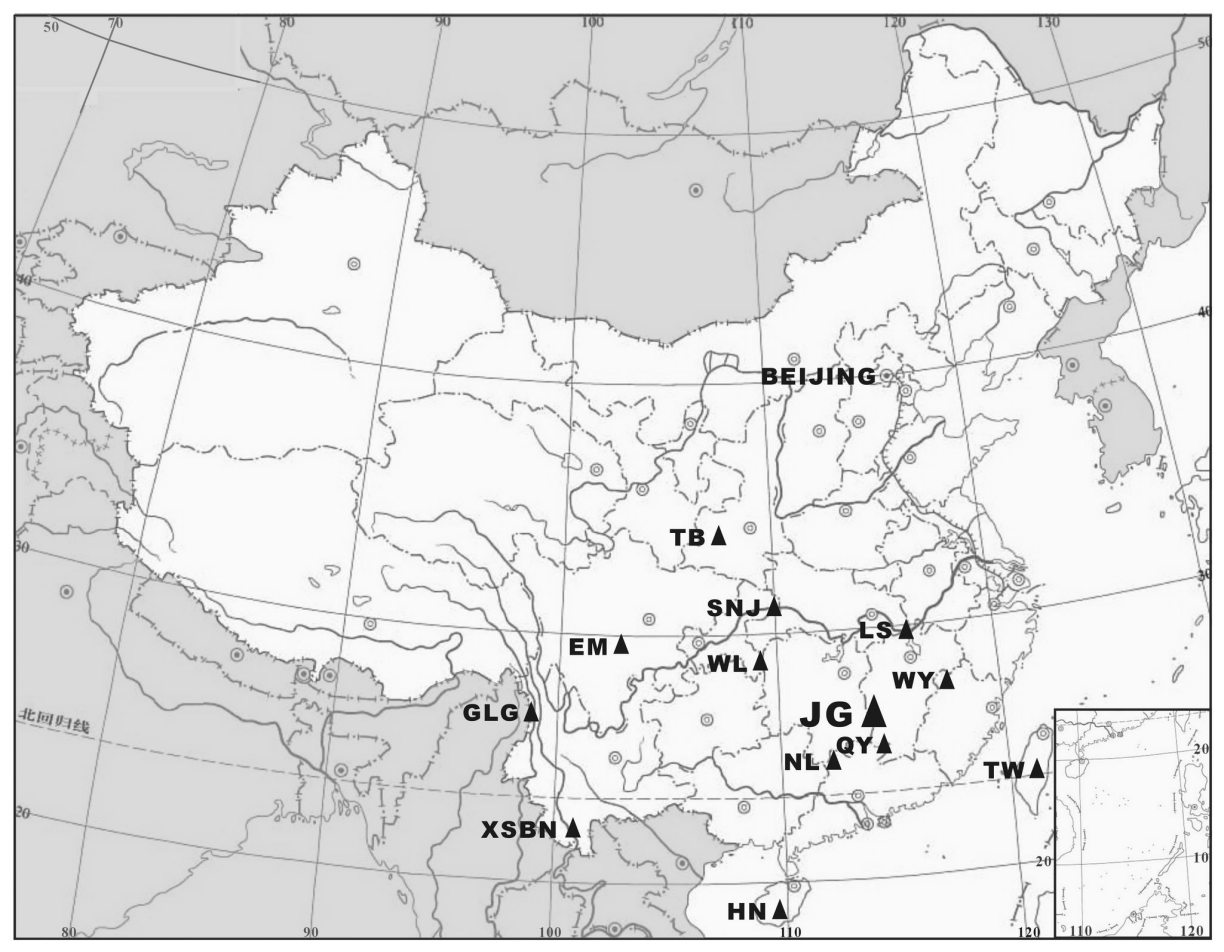
Map showing the locations of Mount Jinggangshan region and other twelve mounts in China. Notes: JG: Mount Jinggangshan region; QY: Mount Qiyunshan; LS: Mount Lushan; NL: Nanling Mountains; WY: Mount Wuyishan region; WL: Wuling Range; SNJ: Mount Shennongxia; EM, Mount Emeishan; TW: Taiwan Mountains; HN: Hainan Mountains; TB: Mount Taibeishan; XSBN: Mount Xishuangbangna; GLG: Mount Gaoligongshan.

The Mount Jinggangshan region, in the central section of the Luoxiao Mountains, has four, linked, nature reserves. It lies between 26° 13'04″-26°52'30″ N and 113° 56′30″-114°22'00″E and covers an area of 480.05 km^2^ (Figure 1). The Luoxiao Mountains is a large north-south trending mountain range that forms the boundary between Jiangxi and Hunan provinces in southeastern China.

### Topography

The Mount Jinggangshan region is deeply cut by deep, V-shaped valleys, resulting in a steep topography. There are two main peaks, Nanfengmian (2,120.4 m above sea level) in Jiangxi Province and Lingfeng (2,122 m) in Hunan Province. The lowest point is 200 m above sea level. The Mount Jinggangshan region has more than 300 peaks above 1,000 m. The relative elevation ranges from 500 to 1,300 m.

### Climate

The Mount Jinggangshan region is under the influence of the monsoon climate and characterized by four distinct seasons and abundant water and heat. Based on the data gathered by the Mount Jinggangshan Weather Bureau from 1971 to 2000, the annual mean temperature is 14.2°C. The hottest month is July, with a mean temperature of 23.9°C and an extreme high temperature of 36.7°C. The coldest month is January, with a mean temperature of 3.4°C and an extreme lowest temperature of −11.0°C. The annual mean sum of radiation ranges from 85 to 105 kcal/cm. The annual cumulative temperature (the sum of daily temperature mean >10°C) is 4,224°C. On average, there are 247.5 fog-free days per year. The annual mean precipitation is 1,889.8 mm with the greatest precipitation of 2,878.8 mm and the lowest precipitation of 1,297.4 mm. The annual mean evaporation capacity is 978.8 mm. The relative humidity is about 85%.

### Soils

There are four major soil types in Mount Jinggangshan region: mountain red, yellow, dark yellow-brown and meadow soil. Among them, mountain yellow soil has the largest distribution area, occurring between 800 and 1,200 m. The second most extensive is mountain red soil, which is often at altitudes below 850 m. Mountain dark yellow-brown soil is mainly between 1,200 and 2,100 m. Mountain meadow soil is mainly between 1,600 m and 1,800 m [1].

### Vegetation

Based on plot investigations and the Vegetation Classification System of China [6], the natural vegetation of Mount Jinggangshan region is divided into 12 types, 82 association groups and 167 associations [5]. Among them, the warm coniferous and broadleaved mixed forests, including *Pinus* *massoniana* mixed forests, *Cunninghamia* *lanceolata* mixed forests, *Amentotaxus* *argotaenia* mixed forests and *Nageia* *nagi* mixed forests, dominate with 28%-30% of the total natural vegetation. Typical evergreen broadleaved forests are also diverse. They include 15 association groups and 39 associations and account for 21.6% of the total natural vegetation, with the main dominant species including *Schima* *superba* , *Schima* *argentea* , *Castanopsis* *sclerophylla* , *Castanopsis* *eyrei* , *Castanopsis* *lamontii* , *Cyclobalanopsis* *glauca* , *Cyclobalanopsis* *sessilifolia* , *Lithocarpus* *glaber* , *Cyclobalanopsis* *multinervis* , *Phoebe* *bournei* , *Phoebe* *hunanensis* , *Machilus* *leptophylla* , *Michelia* *maudiae* , *Michelia* *foveolata* , *Daphniphyllum* *glaucescens* , *Ternstroemia* *gymnanthera* , *Sloanea* *sinensis* , etc.

The next largest type is the ravine or lowland monsoon evergreen broadleaved forests, including 12 association groups and 26 associations. The dominant species comprise *Castanopsis* *fargesii* , *Castanopsis* *tibetana* , *Castanopsis* *fabri* , *Castanopsis* *carlesii* , *Cyclobalanopsis* *fleuryi* , *Altingia* *chinensis* , *Altingia* *gracilipes* , *Elaeocarpus* *japonicus* , *Michelia* *chapensis* , *Manglietia* *fordiana* , *Machilus* *thunbergii* Exbucklandia *tongkinensis*, etc. Generally, these forests are evergreen broadleaved forests that include tree ferns, epiphyllous liverworts, vines and lianas, pteridophytes, epiphytic orchids, trees with buttress roots and strangler vines.

### Geological History

Located in the central section of the Luoxiao Mountains, southeastern Eurasia and southeast of the junction zone of the Yangzi and Cathaysian paleo-plates, Mount Jinggangshan region has experienced a long geological history and undergone complicated evolutionary processes. The region was a basin in the South China Ocean during the Caledonian orogeny of the Neoproterozoic to Eopaleozoic, the margin of the sea during the late Paleozoic, Indosinian orogeny, geological tectonism of Yan mountain and the Himalayan mountain tectonic movement. Cambrian, Ordovician, Devonian and Quaternary strata occur within the Mount Jinggangshan region. The area has been above the sea for more than 20 million years. Through ecological succession, a diversity of life forms have evolved and the distinct vegetation of Mount Jinggangshan region has been formed [1].

## Methods

Firstly, a checklist of the seed plant in Mount Jinggangshan region was prepared. We conducted field surveys and collected specimens in Mount Jinggangshan region from 2009 to 2012, with the permissions of four Nature Reserve Administration. No endangered or protected species were involved. Moreover, we consulted the specimens deposited in the herbarium of the Mount Jinggangshan Natural Reserve and the relevant literatures, such as *Flora of China* [7], *Flora of Jiangxi* [8,9], *List of Jiangxi Seed Plant* [10] and *Species Checklist of Five Provinces in East China* [11].

Secondly, we analyzed the composition and characteristics of the seed plant flora of Mount Jinggangshan region and compared our findings with information from other main mounts in China. Similarity coefficients were calculated according to shared families, genera and species using the formula S = 2c/(a+b) (S is the similarity coefficient between two regions, %; c equals the number of shared taxa; a equals the number of taxa within the region; and b equals the number of taxa within the region). The mounts selected for comparison included Mount Wuyishan region [12-14], Mount Qiyunshan [15], Mount Lushan [16], Wuling Range [17], Mount Emeishan [18], Mount Taibaishan [19], Nanling Mountains [20], Mount Shennongjia [21], Taiwan Mountains [22], Hainan Mountains [23], Mount Xishuangbanna [24] and Mount Gaoligongshan [25].

The Mount Wuyishan region in this paper includes two natural reserves: the Mount Wuyishan Natural Reserve of Fujian Province and the Mount Wuyishan Natural Reserve of Jiangxi Province. The former is situated between 27° 32'36″-27°55'15″ N and 117° 24'12″-118°02'50″ E and covers an area of 565.27 km^2^ [12]. It was listed as a World Natural Heritage site in December, 1999. The latter is situated between 27° 48'11″-28° 00’35″N and 117° 39'30″-117°55'47″ E and covers an area of 160.07 km^2^ [14]. The two natural reserves adjoin each other and share the same main peak, Huanggangshan, therefore, they are regarded as one integrated region. The Mount Wuyishan region is located between 27° 32'36″-28° 00’35″N and 117° 24'12″-118°02'50″ E and covers an area of 725.34 km^2^. The statistics on the seed plants in Mount Jinggangshan region and Mount Wuyishan region were given in Table 1.

**Table 1:** Statistics of the seed plants in Mount Jinggangshan region and Mount Wuyishan region.

Regions/Taxa	Families	Genera	Species	Including infraspecies*	References
Mount Jinggangshan	196	951	1800	1855	[1]
Mount Qixiling	182	651	1427	1464	[3]
Mount Nanfengmian	178	676	1492	1533	[2]
Mount Taoyuandong	187	728	1566	1618	[4]
Total (Mount Jinggangshan region)	210	1003	2958	3187	In this paper
Mount Wuyishan in Fujian Province	189	784	1806	1882	[11]
Mount Wuyishan in Jiangxi Province	193	805	1933	2025	[12]
Total (Mount Wuyishan region)	201	891	2331	2484	In this paper

Notes:The figures in this table may differ from published reports. We have edited the checklists of those mountains to make them uniform at the family, genus and species level based on Zhengyi Wu [26-30]. Infraspecific taxa are considered to be equal to species for statistical purposes.

## Results

### Floristic Composition

Table 2 shows the composition of the seed plant flora of Mount Jinggangshan region. Among them, woody plants are from 350 genera and 1295 species, while herbaceous plants are from 653 genera and 1663 species. Table 3, Table 4, Table 5, Table 6 rank all 210 families in Mount Jinggangshan region based on numbers of genera and species, and contain some information of families, such as the numbers of genera and species in Mount Jinggangshan region [5], in China [31] and in the word [32,33], the numbers of endemic general and endemic species, and Areal-types. The families can be divided into 5 grades according to the numbers of species included (Table 7). The seed plant flora of Mount Jinggangshan region is mainly made up of oligotypic families (100 families) and mesotypic families (57), which account for 74.76% of all families, 55.63% of all genera and 62.43% of all species. Compared with other mounts in rich biodiversity, Mount Jinggangshan region has less single-species families (40), such as Mount Emeishan 47 families, Nanling Mountains 59, Mount Dinghushan 58, Mount Lushan 49, Taiwan Mountain 56, so it goes to show that the flora of Mount Jinggangshan region obtain obvious differentiation.

**Table 2:** Composition of the seed plant flora of Mount Jinggangshan region.

Taxa	Mount Jinggangshan region [6]			Jiangxi Province [8-10]			China [31]		
	family	genus	species	family	genus	species	family	genus	species
Gymnosperms	9	17	23	9	23	35	12	34	230
Angiosperms	201	986	2935	219	1309	4085	325	3166	26046
Dicotyledoneae	170	762	2399						
Monocotyledoneae	31	224	536						
Total (Percent%)	210	1003	2958	228 (92.1)	1332 (75.3)	4120 (71.8)	337 (62.3)	3200 (31.3)	26276 (11.3)

**Table 3:** Ranking of families (with >10 species) in Mount Jinggangshan region based on numbers of genera and species [5,32,33].

Families	Number of genera^1^	GE^2^	Number of species^3^	SE^4^	A^5^	Families	Number of genera	GE	Number of species	SE	A
Poaceae	99/244/668	5	199/1375/10025	31	1	Asclepiadaceae	6/44/180	1	23/220/2200	12	2
Compositae	73/239/1620	1	188/2477/22750	29	1	Hamamelidaceae	12/19/30	2	22/75/95	16	8
Rosaceae	29/56/95		147/1119/2830	70	1	Melastomataceae	8/26/188		22/154/5005	17	2
Cyperaceae	15/37/98		141/673/4350	9	1	Caesalpiniaceae	7/17/165		22/110/2600	6	2
Papilionaceae	46/127/480		131/1100/12000	34	2	Symplocaceae	1/1/1		22/25/320	4	2
Labiatae	33/97/236	2	88/758/7175	37	1	Cucurbitaceae	10/31/118		21/165/845	6	2
Orchidaceae	35/177/880	1	84/1037/21950	14	2	Solanaceae	8/19/102		21/99/2460	2	2
Lauraceae	9/25/50		83/437/2500	50	2	Primulaceae	3/13/20		21/580/800	14	8
Rubiaceae	20/80/600		71/602/1000	19	2	Smilacaceae	2/2/2		21/67/315	6	2
Theaceae	10/15/22	1	60/373/610	43	2	Gesneriaceae	8/57/147	1	20/413/3870	17	2
Fagaceae	6/6/7		58/324/670	30	2	Magnoliaceae	6/11/15	1	20/97/250	11	9
Ranunculaceae	10/40/62		55/739/2525	25	8	Ulmaceae	7/8/16	1	19/47/230	10	2
Scrophulariaceae	20/61/65		54/669/1700	13	1	Acanthaceae	14/63/229		18/175/3500	3	2
Polygonaceae	6/16/43		50/229/1100	6	8	Hydrangeaceae	7/11/17		18/131/190	11	8
Liliaceae	23/50/175	1	48//335/2000	10	1	Styracaceae	6/10/11		17/84/160	11	2
Apiacae	24/101/434	2	47/522/3780	13	8	Tiliaceae	5/10/50		16/79/450	7	3
Euphorbiaceae	13/66/222	1	47/366/5970	11	2	Crassulaceae	3/12/34		16/247/1370	4	1
Aquifoliaceae	1/1/1		47/118/405	27	2	Polygalaceae	2/4/18		16/48/1045	6	1
Ericaceae	5/14/126		43/720/3995	30	8	Araceae	6/28/106		15/193/4025	5	2
Urticaceae	11/25/54		42/238/2625	7	2	Amaranthaceae	4/14/174		15/38/2050	0	1
Caprifoliaceae	4/12/15		42/195/420	3	8	Menispermaceae	8/19/70		14/79/420	5	2
Vitaceae	7/8/14		39/143/850	21	2	Apocynaceae	6/38/415		14/169/4555	6	2
Celastraceae	5/13/69	1	38/213/1300	21	2	Malvaceae	6/17/243		14/68/4225	3	2
Rutaceae	12/28/161	1	34/134/1815	11	2	Aristolochiaceae	3/5/7	1	14/101/350	12	2
Verbenaceae	7/18/30		34/170/1100	13	2	Onagraceae	3/15/24		14/58/650	0	8
Araliaceae	11//25/43	1	32/181/1450	16	2	Elaeagnaceae	1/2/3		14/44/45	9	8
Aceraceae	1/2/3		31/152/200	18	8	Lardizabalaceae	4/7/9		13/33/36	6	8
Cruciferae	15/102/321	1	29/417/3400	1	8	Thymelaeaceae	3/11/48		13/90/891	6	1
Rhamnaceae	6/16/50		29/137/900	17	1	Meliosmaceae	1/1/1		13/35/50	7	3
Myrsinaceae	5/6/41		28/129/1435	6	4	Gentianaceae	3/15/80		12/351/900	3	8
Moraceae	6/9/38		27/161/1100	5	2	Dioscoreaceae	1/1/3		12/80/805	3	2
Violaceae	1/4/20		26/125/800	8	1	Boraginaceae	7/47/148	3	11/186/2740	5	8
Caryophyllaceae	13/28/86		24/294/2200	2	1	Loranthaceae	6/8/68		11/50/950	5	2
Oleaceae	7/13/24		24/180/615	18	2	Campanulaceae	5/15/84		11/136/2380	4	8
Actinidiaceae	2/2/3	1	24/53/355	18	14	Saxifragaceae	4/13/29		11/281/630	5	8

Notes: ^1^ Number of genera in Mount Jinggangshan region/number in China /number in the world; ^2^ Number of genera endemic to China; ^3^ Number of species in Mount Jinggangshan region/number in China /number in the world; ^4^ Number of species endemic to China; ^5^ Areal-types of families of the seed plants in Mount Jinggangshan region.

**Table 4:** Ranking of families (with 5-10 species) in Mount Jinggangshan region based on numbers of genera and species.

Families	Number of genera^1^	GE^2^	Number of species^3^	SE^4^	A^5^	Families	Number of genera	GE	Number of species	SE	A
Convolvulaceae	7/21/57		10/115/1600	0	1	Utriculariaceae	1/2/3		7/19/320	1	1
Lythraceae	4/11/25		10/48/550	1	1	Hydrocharitaceae	5/9/18		6/22/116	0	2
Anacardiaceae	3/15/70		10/51/985	3	2	Juglandaceae	5/7/9	1	6/27/50	2	8
Buxaceae	3/3/4		10/22/70	8	2	Sterculiaceae	4/20/68		6/80/1100	1	2
Cornaceae	3/5/12		10/51/100	6	8	Flacourtiaceae	3/10/80	1	6/21/500	1	2
Corylaceae	2/4/4		10/30/69	5	8	Chenopodiaceae	2/49/100		6/198/1400	0	1
Schisandraceae	2/2/3		10/29/92	7	9	Geraniaceae	2/2/7		6/68/805	0	1
Balsaminaceae	1/2/2		10/191/1000	8	2	Pinaceae	4/10/11	1	6/87/210	5	8
Hypericaceae	1/4/10		10/54/300	2	1	Podophyllaceae	2/2/2		6/7/7	5	9
Commelinaceae	6/13/40		9/53/652	0	2	Trilliaceae	2/2/4		6/10/50	1	9
Berberidaceae	2/2/14		9/245/701	9	9	Eriocaulaceae	1/1/10		6/45/1160	1	2
Begoniaceae	1/1/2		9/90/1401	8	2	Potamogetonaceae	1/1/2		6/30/102	0	1
Fumariaceae	1/7/16		9/215/450	1	8	Taxaceae	4/4/6	1	5/13/23	2	8
Chloranthaceae	2/3/4		8/16/75	4	2	Lemnaceae	3/3/6		5/7/30	0	1
Ebenaceae	1/1/4		8/58/548	4	2	Meliaceae	3/6/52		5/10/261	1	2
Sabiaceae	1/1/3		8/26/100	7	7	Staphyleaceae	3/3/3		5/15/45	2	3
Vacciniaceae	1/2/22		8/87/400	7	1	Zingiberaceae	3/20/49		5/145/1188	1	7
Naucleaceae	5/8/11		7/28/143	0	4	Lobeliaceae	2/3/20		5/24/600	1	2
Alismataceae	2/5/12		7/12/81	0	1	Valerianaceae	2/4/17		5/40/315	1	8
Elaeocarpaceae	2/2/12		7/52/625	1	2	Alangiaceae	1/1/1		5/8/17	2	2
Juncaceae	2/2/7		7/80/430	0	1	Clethraceae	1/1/2		5/16/76	1	3
Salicaceae	2/2/55		7/228/1010	7	8	Oxalidaceae	1/2/6		5/13/770	0	1
Pittosporaceae	1/1/9		7/34/200	2	4	Piperaceae	1/4/5		5/54/3600	3	2

Notes: ^1^ Number of genera in Mount Jinggangshan region/number in China /number in the world; ^2^ Number of genera endemic to China; ^3^ Number of species in Mount Jinggangshan region/number in China /number in the world; ^4^ Number of species endemic to China; ^5^ Areal-types of families of the seed plants in Mount Jinggangshan region.

**Table 5:** Ranking of families (with 2-4 species) in Mount Jinggangshan region based on numbers of genera and species.

Families	Number of genera^1^	GE^2^	Number of species^3^	SE^4^	A^5^	Families	Number of genera	GE	Number of species	SE	A
Cupressaceae	4/8/29		4/31/140	2	8	Iteaceae	1/1/2		3/12/18	1	9
Papaveraceae	3/11/41	1	4/58/760	2	8	Myrtaceae	1/10/131		3/91/4625	2	2
Haloragaceae	2/2/8		4/7/145	0	1	Pyrolaceae	1/4/14		3/31/40	1	8
Iridaceae	2/5/67		4/44/1800	1	2	Stilaginaceae	1/1/1		3/17/170	1	4
Loganiaceae	2/5/13		4/15/420	2	2	Trapaceae	1/1/1		3/5/30	0	10
Annonaceae	1/22/129		4/122/2220	2	4	Guttiferae	2/5/27		2/210/450	0	2
Aucubaceae	1/1/1		4/11/11	1	14	Hypoxidaceae	2/3/8		2/8/160	0	2
Balanophoraceae	1/2/7		4/18/50	1	2	Nyssaceae	2/2/5	1	2/7/22	1	9
Cephalotaxaceae	1/1/1		4/7/28	3	14	Portulacaceae	2/3/36		2/7/395	0	1
Droseraceae	1/2/3		4/7/115	0	1	Santalaceae	2/7/44		2/32/935	0	2
Illiciaceae	1/1/1		4/26/50	4	9	Saururaceae	2/3/4		2/4/6	0	9
Philadelphaceae	1/1/1		4/15/75	4	9	Acoraceae	1/1/1		2/4/4	0	8
Phytolaccaceae	1/2/18		4/7/65	1	2	Amaryllidaceae	1/5/59		2/23/800	0	2
Plantaginaceae	1/1/47		4/13/1350	0	1	Callitrichaceae	1/1/1		2/4/25	1	1
Viscaceae	1/1/2		4/11/70	1	1	Ceratophyllaceae	1/1/1		2/5/7	0	1
Sapindaceae	3/25/135	1	3/51/1580	2	2	Cleomaceae	1/1/10		2/3/300	0	2
Simarubaceae	3/4/19		3/12/95	1	2	Dipsacaceae	1/4/11		2/20/290	0	10
Betulaceae	2/2/6		3/37/145	1	8	Myricaceae	1/1/3		2/4/57	0	1
Mimosaceae	2/11/56		3/65/2800	0	2	Olacaceae	1/4/14		2/8/103	1	2
Monotropaceae	2/4/12		3/5/21	0	8	Orobanchaceae	1/10/99		2/40/2060	0	8
Pontederiaceae	2/2/9		3/5/33	0	2	Parnassiaceae	1/1/2		2/36/51	0	8
Buddlejaceae	1/1/7		3/29/100	1	2	Podocarpaceae	1/4/17		2/20/125	0	8
Calycanthaceae	1/2/5		3/4/11	2	9	Proteaceae	1/2/80		2/21/1600	1	2
Cuscutaceae	1/1/1		3/10/170	0	1	Sambucaceae	1/1/1		2/5/20	1	8
Daphniphyllaceae	1/1/1		3/12/25	0	7	Stachyuraceae	1/1/1		2/8/10	1	14
Ehretiaceae	1/1/1		3/12/50	0	2	Stemonaceae	1/1/4		2/8/27	1	5
Helwingiaceae	1/1/1		3/5/8	0	14	Typhaceae	1/1/1		2/10/11	0	1

Notes: ^1^ Number of genera in Mount Jinggangshan region/number in China /number in the world; ^2^ Number of genera endemic to China; ^3^ Number of species in Mount Jinggangshan region/number in China /number in the world; ^4^ Number of species endemic to China; ^5^ Areal-types of families of the seed plants in Mount Jinggangshan region.

**Table 6:** Ranking of families (with 1 species) in Mount Jinggangshan region based on numbers of genera and species.

Families	Number of genera^1^	GE^2^	Number of species^3^	SE^4^	A^5^	Families	Number of genera	GE	Number of species	SE	A
Alliaceae	1/3/13		1/112/795	0	1	Nageiaceae	1/1/1		1/5/5	0	7
Asparagaceae	1/1/1		1/24/300	0	5	Najadaceae	1/1/1		1/4/50	0	1
Bignoniaceae	1/14/110		1/43/800	0	4	Nandinaceae	1/1/1		1/1/1	0	14
Bischofiaceae	1/1/1		1/2/2	1	7	Nelumbonaceae	1/1/1		1/1/2	0	9
Bretschneideraceae	1/1/1	1	1/1/1	1	15	Nymphaeaceae	1/2/5		1/8/48	0	1
Cabombaceae	1/1/2		1/1/6	0	1	Palmae	1/19/189		1/95/2361	0	2
Cannabaceae	1/2/2		1/3/3	0	8	Passifloraceae	1/2/16		1/22/605	1	2
Capparaceae	1/4/16		1/30/480	0	2	Penthoraceae	1/1/1		1/1/2	0	9
Ellisiophyllaceae	1/1/1		1/1/1	0	7	Periplocaceae	1/1/1		1/4/12	1	2
Erythroxylaceae	1/1/4		1/3/240	0	2	Phrymataceae	1/1/1		1/1/1	0	9
Eucommiaceae	1/1/1	1	1/1/1	1	15	Pistaciaceae	1/1/1		1/3/10	1	12
Euryalaceae	1/1/5		1/1/95	0	14	Samydaceae	1/2/17		1/18/400	0	2
Ginkgoaceae	1/1/1	1	1/1/1	1	15	Sargentodoxaceae	1/1/1	1	1/1/1	1	15
Gnetaceae	1/1/1		1/7/30	0	2	Sparganiaceae	1/1/1		1/5/14	0	8
Grossulariaceae	1/1/1		1/45/150	0	8	Spigeliaceae	1/2/2		1/6/8	0	2
Hippocastanaceae	1/1/2		1/8/32	1	8	Taccaceae	1/2/2		1/4/12	0	2
Hydrocotylaceae	1/1/1		1/2/2	0	2	Tapisciaceae	1/1/2	1	1/2/5	1	15
Menyanthaceae	1/2/5		1/4/58	0	1	Taxodiaceae	1/5/30	1	1/7/130	0	9
Molluginaceae	1/2/9		1/6/87	0	2	Zannichelliaceae	1/1/6		1/1/20	0	1
Musaceae	1/3/3		1/7/35	0	4	Zygophyllaceae	1/5/26		1/27/285	0	1

Notes: ^1^ Number of genera in Mount Jinggangshan region/number in China /number in the world; ^2^ Number of genera endemic to China; ^3^ Number of species in Mount Jinggangshan region/number in China /number in the world; ^4^ Number of species endemic to China; ^5^ Areal-types of families of the seed plants in Mount Jinggangshan region.

**Table 7:** Statistics of the family size from the seed plant flora of Mount Jinggangshan region.

Taxa	Single-species families (1 species)	Oligotypic families (2-10 species)	Mesotypic families (11-50 species)	Pluritypic families (51-100 species)	Macrotypic families (>100 species)
Gymnosperms	4 (4:4)^1^	5 (14:21)			
Angiosperms	36 (36:36)	95 (173:462)	57 (371:1361)	8 (143:553)	5 (262:806)
Total	40 (40:40)	100 (187:483)	57 (371:1361)	8 (143:553)	5 (262:806)
Percent of total (%)	19.05/3.99/1.35^2^	47.62/18.64/16.33	27.14/36.99/46.01	3.81/14.26/18.70	2.38/26.12/27.25

Notes: ^1^ Number of families (number of genera included in the families : number of species included in the families); ^2^ Percent of all 210 families (percent of all 1003 genera : percent of all 2958 species).

The typical families in Mount Jinggangshan region are Fagaceae, Lauraceae, Theaceae, Hamamelidaceae, Magnoliaceae, Ericaceae, Styracaceae, Aquifoliaceae, Elaeocarpaceae, Aceraceae, Daphniphyllaceae, Hydrangeaceae, Rosaceae, Symplocaceae, Euphorbiaceae, Pinaceae, Taxodiaceae, Cupressaceae and Taxaceae. Other families with biogeographical implication are Bretschneideraceae, Tapisciaceae, Sargentodoxaceae, Eucommiaceae, Ginkgoaceae, Nyssaceae, Stachyuraceae, Helwingiaceae, Aucubaceae, Berberidaceae, Corylaceae, Cephalotaxaceae, Actinidiaceae and Schisandraceae, etc.

### Floristic Geographic Elements

According to the concept of families proposed by Zhengyi Wu [28,29,30], the family areal-types of spermatophyte flora in Mount Jinggangshan region can be divided into 12 types (Table 8, Table 3, Table 4, Table 5, Table 6), which are further grouped into three categories: Cosmopolitan, Tropical and Temperate.

**Table 8:** Areal-types of families from the seed plant flora of Mount Jinggangshan region (JGR).

Areal-types of families	Number of families in JGR / in China	Percent of total non-cosmopolitan families in JGR (%)
1. Cosmopolitan	42/50	-—
2. Pantropic	81/120	48.21
3. Tropical Asian and tropical American disjunct	5/11	2.98
4. Old World Tropic	7/17	4.17
5. Tropical Asia to Tropical Australia	2/10	1.19
6. Tropical Asia to tropical Africa	0 / 7	0
7. Tropical Asia (Indo-Malaysia)	6/20	3.57
8. North temperate	38/47	22.62
9. East Asian and North American disjunct	14/17	8.33
10. Old World temperate	2/6	1.19
11. Temperate Asia	0 / 0	0
12. Mediterranean region, western to central Asia	1/8	0.60
13. Central Asia	0 / 1	0
14. East Asia	7/18	4.17
15. Endemic to China	5/8	2.98
Total	210/337	100

The genera can be divided into 5 categories according to size (Table 9). Based on the generic distribution concept proposed by Zhengyi Wu [26,27], the 1,003 seed plant genera can be divided into 14 types and 17 sub-types (Table 10). Those types and sub-types can be further sorted into three groups: cosmopolitan, 79 genera (7.88% of total, including 456 species); tropical, 452 genera (48.92% of non-cosmopolitan genera, including 1168 species); and temperate, 472 genera (51.08% of non-cosmopolitan genera, including 1278 species). Temperate genera contain 44 genera endemics to China (4.76% of non-cosmopolitan genera, including 56 species). The larger cosmopolitan genera include *Cyperus* (including 48 species), *Rubu*s (45), *Carex* (42), *Polygonum* (37), *Viola* (26), *Lysimachia* (19), *Clematis* (24), *Polygala* (14), *Salvia* (11), *Rhamnus* (10) and *Ranunculus* (9). Among no-cosmopolitan genera, 51 genera with more than 10 species include 971 species, accounting for 32.83% of all species. For example, *Ilex* has 47 species, *Rhododendron* 31, *Acer* 31, *Symplocos* 22, *Euonymus* 21, *Camellia* 20, *Eurya* 19, *Litsea* 17, *Cinnamomum* 14, *Machilus* 13, *Neolitsea* 11, *Lithocarpus* 15, *Cyclobalanopsis* 14, *Castanopsis* 13, *Ficus* 16, *Callicarpa* 15, *Photinia* 14, *Meliosma* 13 and *Styrax* 11. The genera including the dominant species in arborous layer, in shrub layer and in herbaceous layer in Mount Jinggangshan region are listed in Table 11.

**Table 9:** Statistics of the genus size from the seed plant flora of Mount Jinaggangshan region.

Taxa	Single-species genera^1^ (1 species)	Oligotypic genera (2-5 species)	Mesotypic genera (6-10 species)	Pluritypic genera (11-20 species)	Macrotypic genera (>21 species)
Gymnosperms	13 (13)^2^	5 (12)			
Angiosperms	452 (452)	396 (1155)	86 (640)	37 (529)	14 (442)
Total	465 (465)	401 (1167)	86 (640)	37 (529)	14 (442)

Notes: ^1^ Single-species genera include monotypic genus containing only one species in the world. There are 63 monotypic genera in the Mount Jinggangshan region; ^2^ Number of genera (number of species included in the genera).

**Table 10:** Areal-types of genera from the seed plant flora of Mount Jinggangshan region (JGR).

Areal-types of genera	Number of genera in JGR / in China	Percent of the total non-cosmopolitan genera in JGR (%)
1. Cosmopolitan	79/107	-—
2. Pantropic	164/304	17.75
2-1.Tropical Asia, Australasia (to New Zealand) and Central to South America (or Mexico) disjunct	10/21	1.08
2-2.Tropical Asia, Africa and Central to South America disjunct	8/32	0.87
3. Tropical Asian and tropical American disjunct	17/78	1.84
4. Old World Tropical	54/150	5.84
4-1.Tropical Asia, Africa (or East Africa, Madagascar) and Australasia disjunct	12/27	1.30
5. Tropical Asia to Tropical Australia	42/154	4.55
5-1.Chinese (southwest) Subtropical and New Zealand disjunct	1/2	0.11
6. Tropical Asia to tropical Africa	29/145	3.14
6-2.Tropical Asia and East Africa or Madagascar disjunct	3/8	0.32
7. Tropical Asia (Indo-Malaysia)	80/460	8.66
7-1.Java (or Sumatra), Himalaya to south and southwest China disjunct or dispersed	12/31	1.30
7-2.Tropical India to south China (particularly southern Yunnan)	6/53	0.65
7-3.Burma, Thailand to southwest China	4/36	0.43
7-4.Vietnam (or Indochina) to south China (or southwest China)	10/66	1.08
8. North temperate	113/193	12.23
8-4.North Temperate and south temperate disjunct	31/78	3.35
8-5.Eurasia and South America temperate disjunct	1/8	0.11
9. East Asia and North America disjunct	75/122	8.12
9-1.East Asia and Mexicao disjunct	1/2	0.11
10.Old World temperate	41/119	4.44
10-1.Mediterranean, western Asia (or central Asia) and East Asia disjunct	9/30	0.97
10-2.Mediterranean and Himalayan disjunct	1/7	0.11
10-3.Eurasia and southern Africa (sometimes Australasia also) disjunct	4/18	0.43
11. Temperate Asia	14/64	1.52
12. Mediterranean region, western to central Asia	3/133	0.32
12-3.Mediterranen to temperate-tropical Asia, Australasia and South America disjunct	2/7	0.22
13. Central Asia	1/73	0.11
14. East Asia	60/75	6.49
14-1.Sino-Himalayan (SH)	21/142	2.27
14-2.Sino-Japanese (SJ)	51/100	5.52
15. Endemic to China*	44/251	4.76
Total	1003/3200	100

Notes: * The genera endemic to China include *Ginkgo* , *Cunninghamia* , *Pseudotaxus* , *Parakmeria* , *Tsoogiodendron*, *Sargentodoxa* , *Saruma*, *Eomencon*, *Yinshania*, *Poliothyrsis* , *Tutcheria* , *Clematoclethra* , *Speranskia* , *Chimonanthus* , *Fortunearia* , *Semiliquidambar* , *Eucommia* , *Pteroceltis* , *Monimopetalum* , *Poncirus* , *Eurycorymbus* , *Bretschneidera* , *Tapiscia* , *Cyclocarya* , *Camptotheca*, *Metapanax*, *Tetrapanax* , *Changium* , *Dickinsia* , *Melliodendron* , *Biondia* , *Sheareria* , *Sinojohnstonia* , *Thyrocarpus* , *Schnabelia* , *Bostrychanthera* , *Hanceola* , *Speirantha* , *Changnienia* , *Indocalamus* , *Gelidocalamus* , *Oligostachyum* , *Omphalotrigonotis* , *Emmenopterys* .

**Table 11:** The genera including the dominant species in arborous layer, in shrub layer and in herbaceous layer of the vegetation of Mount Jinggangshan region.

**genera**	**arborous layer**	**shrub layer**	**herbaceous layer**
**Tropical**	*Cyclobalanopsis* , *Lithocarpus* , *Ternstroemia* , *Cleyera* , *Schima*, *Camellia* , *Cinnamomum* , *Machilus* , *Phoebe*, *Neolitsea* , *Exbucklandia* , *Sycopsis* , *Distylium* , *Michelia*, *Manglietia* , *Styrax*, *Alniphyllum* *, * *Elaeocarpus* , *Symplocos* , *Ficus*, *Dendropanax* , *Sapium*, *Fokienia* , *Amentotaxus*	*Eurya*, *Maesa*, *Ardisia* , *Turpinia* , *Sageretia* , *Tarenna* , *Smilax*, *Clethra* , *Lasianthus* , *Psychotria* , *Bambusa* , *Helicteres* , *Mallotus* , *Embelia* , *Gardenia* , *Smithia* , *Melastoma* , *Myrsine* , *Adina*, *Toddalia* , *Lindera* , *Kadsura*	*Impatiens* , *Osbeckia* *, * *Phyllagathis* , *Elatostema* , *Pellionia* , *Didymocarpus* , *Ophiorrhiza* , *Elephantopus* , *Sesbania* *, * *Eupatorium* , *Pollia*, *Lycianthes* , *Alpinia* , *Paraphlomis* , *Floscopa* , *Brachiaria* , *Cyrtococcum* , *Heteropogon* , *Ichnanthus* , *Miscanthus* , *Thysanolaena* , *Pennisetum* , *Sporobolus* , *Coelogyne* , *Bulbostylis* , *Neyraudia* , *Fimbristylis*
**Temperate**	*Quercus* , *Fagus*, *Castanopsis* , *Castanea* , *Rhododendron* , *Lyonia*, *Cerasus* , *Sorbus*, *Carpinus* , *Dendrobenthamia*, *Cornus*, *Acer*, *Alnus*, *Osmanthus* , *Magnolia* , *Illicium* , *Halesia* , *Stewartia* , *Liquidambar* , *Pterocarya* *, Pinus*, *Abies*, *Taxus*, *Tsuga*	*Viburnum* , *Elaeagnus* , *Rosa*, *Berberis* , *Fraxinus* , *Vaccinium* , *Sambucus* , *Hydrangea* , *Toxicodendron* , *Lyonia*, *Pieris*, *Actinidia* , *Bredia*, *Deutzia* , *Enkianthus* , *Holboellia* , *Pleioblastus*	*Cimicifuga* , *Eomecon* , *Draba*, *Sedum*, *Saxifraga* , *Fagopyrum* , *Circaea* , *Bredia*, *Geum* , *Habenaria* , *Oenanthe* , *Valeriana* , *Artemisia* , *Inula*, *Kalimeris* , *Swertia* , *Trigonotis* , *Bothriospermum* , *Pedicularis* , *Hosta* , *Veronicastrum* , *Oreocharis* , *Prunella* , *Aspidistra* , *Liriope* , *Acorus*, *Arundinella* , *Aster* , *Deyeuxia* , *Spodiopogon* , *Triarrhena*

### Comparison with Other Twelve Main Mounts in China

Table 12 shows similarity coefficients between Mount Jinggangshan region and other twelve main mounts in China. Among them, the flora of Mount Jinggangshan region is most similar to Mount Wuyishan region’s, as one would expect from their close proximity. The similarities at family, genus and species level are 93.4%, 85.1% and 71.6%, respectively. Next are Mount Qiyunshan (family 89.8% /genus 81.4% /species 67.5%) and Mount Lushan (92.5%/81.3%/63.3%). The above mountains are all in eastern China. The third most similar regions are the Nanling Mountains (south of Mount Jinggangshan region) (90.4%/77.6%/61.6%) and the Wuling Range (west of Mount Jinggangshan) (93.1%/81.0%/57.6%).

**Table 12:** Similarity coefficients at family, genus and species between Mount Jinggangshan region and other twelve main mounts in China.

Mount		JG	WY	QY	LS	NL	WL	SNJ	EM	TW	HN	TB	XSBN	GLG
Area (km^2^)		480	725	110	292	563	10000	705	154	35990	33000	563	2418	4046
	F^1^	210	201	182	192	219	213	181	202	246	244	156	236	215
	G^2^	1003	891	790	757	996	964	780	869	1251	1247	633	1217	1025
	S^3^	2958	2331	2152	1732	2713	3040	2155	2411	3378	3369	1759	3767	3798
WY	F^4^	192/93.4												
	G^5^	809/85.4												
	S^6^	1814/68.6												
QY	F	176/89.8	170/88.8											
	G	730/81.4	668/74.5											
	S	1724/67.5	1409/62.9											
LS	F	186/92.5	179/91.1	164/87.7										
	G	715/81.3	645/78.3	580/75.0										
	S	1484/63.3	1244/61.2	1056/54.4										
NL	F	194/90.4	188/89.5	179/89.3	176/85.6									
	G	776/77.6	704/74.6	720/80.6	591/67.4									
	S	1746/61.6	1427/56.6	1560/64.1	993/44.7									
WL	F	197/93.1	197/95.2	174/88.1	184/90.9	190/88.0								
	G	797/81.0	724/78.1	649/74.0	645/75.0	713/72.8								
	S	1726/57.6	1352/50.3	1241/47.8	1126/47.2	1337/46.5								
SNJ	F	168/85.9	167/87.4	151/83.2	159/85.3	165/82.5	179/90.9							
	G	618/69.3	576/68.9	497/63.3	533/69.4	537/60.5	658/75.5							
	S	1079/42.2	873/38.9	730/33.9	821/42.2	751/30.9	1346/51.8							
EM	F	182/88.4	180/89.3	164/85.4	172/87.3	181/86.0	159/76.6	169/88.3						
	G	677/72.3	629/71.5	580/69.9	555/68.3	627/67.2	715/78.0	602/73.0						
	S	1073/40.0	839/35.4	802/35.2	731/35.3	869/33.9	1353/49.6	1024/44.9						
TW	F	196/86.0	186/83.2	171/79.9	178/81.3	199/85.6	192/83.7	164/76.8	182/81.3					
	G	752/66.7	677/63.2	624/61.1	586/58.4	726/64.6	689/62.2	528/52.0	632/59.6					
	S	949/30.0	781/27.4	707/25.6	633/24.8	829/27.2	714/22.2	416/15.0	535/18.5					
HN	F	175/77.1	167/75.1	168/78.9	159/72.9	190/82.1	140/61.3	144/67.8	161/72.2	207/84.5				
	G	600/53.3	553/51.7	547/53.7	450/44.9	664/59.2	526/47.6	358/35.3	470/44.4	799/64.0				
	S	905/28.6	747/26.2	834/30.2	482/18.9	1103/36.3	605/18.9	281/10.2	444/15.4	899/26.6				
TB	F	142/77.6	139/77.9	125/74.0	140/80.5	136/72.5	133/72.1	144/85.5	145/81.0	138/68.7	120/60.0			
	G	468/57.2	428/56.2	362/50.9	421/60.6	374/45.9	473/59.2	524/74.2	445/59.3	399/42.4	247/26.3			
	S	653/27.7	518/25.3	414/21.2	553/31.7	401/17.9	748/31.2	951/48.6	605/29.0	251/9.8	162/6.3			
XSBN	F	183/82.1	173/79.2	171/81.8	165/77.1	196/81.3	147/65.5	150/71.9	169/77.2	203/84.2	212/88.3	128/65.3		
	G	611/55.0	548/52.0	541/53.9	443/44.9	659/59.6	569/52.2	399/40.0	526/50.4	755/61.2	863/70.0	279/30.2		
	S	804/23.9	564/18.5	674/22.8	464/16.9	882/27.2	699/20.5	340/11.5	604/19.6	646/18.1	1304/36.5	189/6.8		
GLG	F	185/87.1	178/85.6	168/84.6	170/83.5	186/85.7	155/72.4	167/84.3	187/89.7	193/83.7	175/76.3	143/77.1	182/80.7	
	G	649/64.0	588/61.4	556/61.3	517/58.0	638/63.1	678/68.2	566/62.7	660/69.7	671/59.0	557/49.0	433/52.2	661/59.0	
	S	741/21.9	562/18.3	554/18.6	474/17.1	656/20.2	910/26.6	659/22.1	947/30.5	510/14.2	525/14.7	456/16.4	1110/29.3	

Notes: ^1^ Number of families in the mount; ^2^ Number of genera in the mount; ^3^ Number of species in the mount; ^4^ Number of shared families/coefficient of similarity (%); ^5^ Number of shared genera/coefficient of similarity (%); ^6^ Number of shared species/coefficient of similarity (%). JG: Mount Jinggangshan region; WY: Mount Wuyishan region; QY: Mount Qiyunshan; LS: Mount Lushan; NL: Nanling Mountains; WL: Wuling Range; SNJ: Mount Shennongxia; EM, Mount Emeishan; TW: Taiwan Mountains; HN: Hainan Mountains; TB: Mount Taibeishan; XSBN: Mount Xishuangbangna; GLG: Mount Gaoligongshan.

Mount Jinggangshan region is at the northern distribution limit of Gnetaceae and Samydaceae. They occur in Mount Jinggangshan region and Nanling Mountains, but not in Mount Wuyishan region, Wuling Range or Mount Lushan. The Nageiaceae, Capparaceae and Erythroxylaceae, mainly in southern China, are also in Mount Jinggangshan region and Mount Wuyishan region, but not Mount Lushan and Wuling Range. The Zygophyllaceae, mainly in northern dry areas, is in Mount Jinggangshan, but not Mount Qiyunshan, Nanling Mountains or Mount Wuyishan region. Mount Jinggangshan region marks the southernmost distribution of Zygophyllaceae.

Some important tropical or south tropical genera such as *Tsoongiodendron* , *Exbucklandia* , *Altingia* , *Disanthus* , *Semiliquidambar* , *Cryptocarya* , *Anneslea* , *Garcinia* , *Pithecellobium* , *Heteropanax* , *Psychotria* , *Passiflora* , *Blastus* and *Bredia* are mainly in southern China. They occur in Mount Jinggangshan region, but not Mount Lushan or Wuling Range.

Some typical genera in central China, such as *Saruma*, *Clematoclethra* and *Dickinsia* [34], occur in Mount Jinggangshan region, but not Mount Wuyishan region, Mount Qiyunshan or Nanling Mountains. Some genera endemic to central and eastern China, including *Eucommia* , *Bretschneidera* , *Sargentodoxa* , *Pseudotaxus* , *Cyclocarya* , *Changnienia* , *Eomecon* , *Changium* , *Fortunearia* , *Sheareria* , are also in Mount Jinggangshan. The following genera are mainly in Mount Jinggangshan region and eastern China: *Ginkgo* , *Cunninghamia* , *Bretschneidera* , *Monimopetalum* , *Hilliella*, *Gelidocalamus* , *Speirantha* .

**Figure 2:**
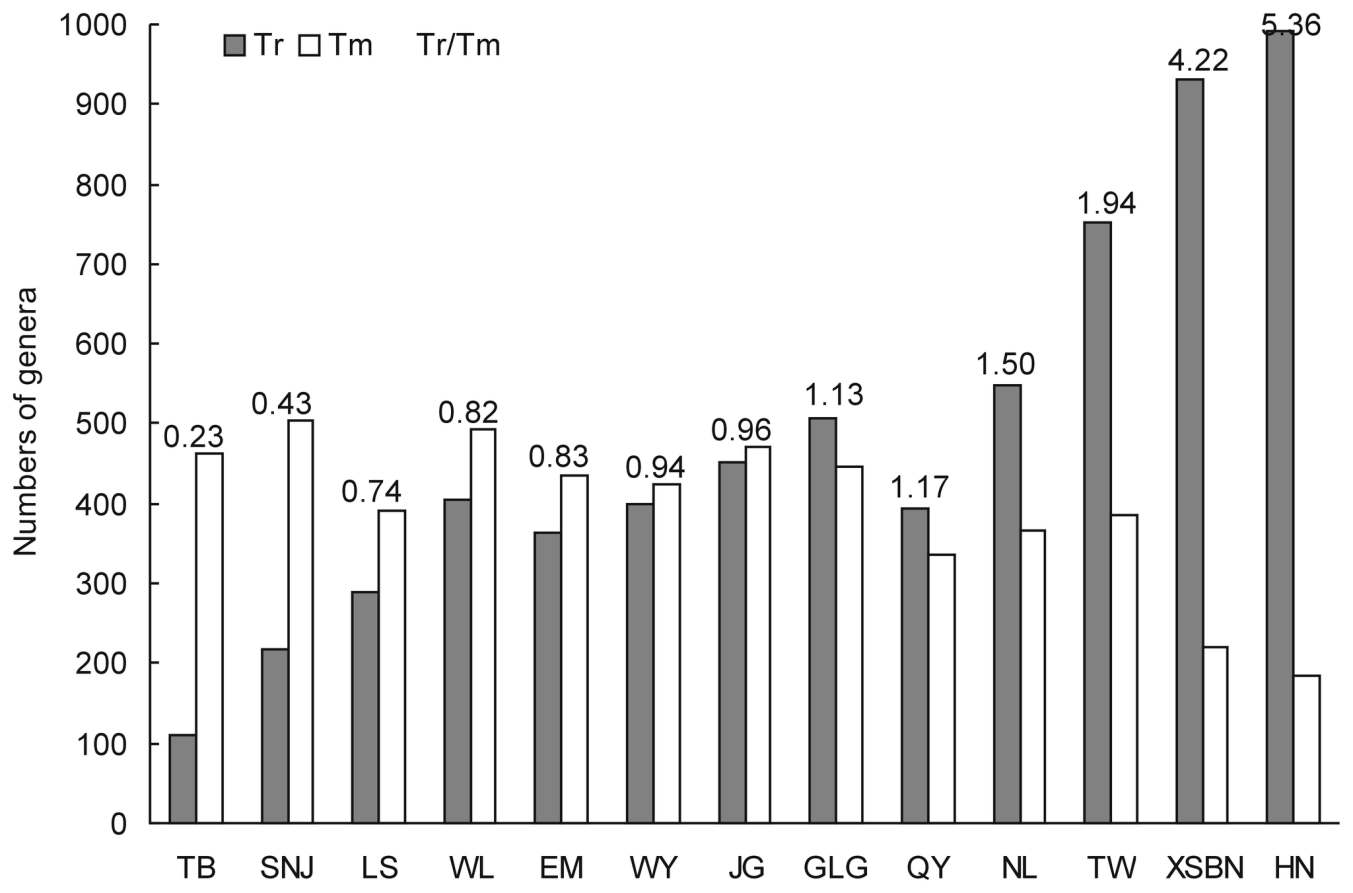
Numbers and ratios of the tropical genera and the temperate genera in Mount Jinggangshan region and other twelve mounts in China.

From Figure 1 and Figure 2, it can be seen that tropical genera (Tr) increase and temperate genera (Tm) decrease from north to south and from west to east. The rate of change is expressed by Tr/Tm value. For example, Mount Jinggangshan region has 452 tropical genera and slightly less than 472 temperate genera, so Tr/Tm=452/472=0.96. The Mount Wuyishan region has 400 tropical genera and slight less than 425 temperate genera, so Tr/Tm=0.94.

## Discussion and Conclusions

The flora of the Mount Jinggangshan region includes abundant primitive gymnosperms and angiosperms. Among them are 9 families of gymnosperms with 17 genera and 23 species. They account for 75.0%, 50.0% and 10.0% of wild gymnosperms (12 families, 34 genera and 230 species) in China respectively, and 69.2%, 23.9% and 2.9% of all gymnosperms (13 families, 71 genera and 800 species) in the world. *Ginkgo biloba* can be traced back to the Permian. *Amentotaxus* *argotaenia* , *Fokienia* *hodginsii* and *Podocarpus* *macrophyllus* were widely distributed in Cretaceous. *Abies* *beshanzuensis* var. *ziyuanensis* , endemic to China and critically endangered (<500 individuals in 2013), is looked as an evidence that *Abies* migrated from north to south and from high elevation to low elevation during the Quaternary ice age and withdraw to north and to higher elevation in post glacial [35]. The Mount Jinggangshan region is the largest distribution area of *Abies* *beshanzuensis* var. *ziyuanensis* (<350 individuals in 2013) and the lowest distribution area of *Abies* at the same latitude in the world.

To angiosperms, there are plentiful primitive representatives of the ancient families, such as *Tsoongiodendron* *odorum* , *Manglietia* *fordiana* and *Magnolia* *officinalis* , belonging to Magnoliaceae, and *Exbucklandia* *tonkinensis* , *Hamamelis* *mollis* and *Fortunearia* *sinensis* , belonging to Hamamelidaceae. The fossils of *Exbucklandia* (in the Paleocene in North America and China), *Fortunearia* (in the Oligocene in German and Pliocene in Japan), and *Hamamelis* (in Cretaceous in Sweden and Eocene in China) have been discovered [32,36-38]. Other primitive taxa include *Disanthus* *cercidifolia* subsp. *longipes* , *Eucommia* *ulmoides* , *Cyclocarya* *paliurus* , *Sargentodoxa* *cuneata* , *Bretschneidera* *sinensis* , *Camptotheca* *acuminata* , and *Eurycorymbus* *cavaleriei* .

As a refugium, there are many communities consisting of relict species as the dominant species, such as *Amentotaxus* *argotaenia* , *Cunninghamia* *lanceolata* , *Fokienia* *hodginsii* , *Nageia* *nagi* , *Pseudotaxus* *chienii* , *Taxus* *wallichiana* var. *mairei* , *Tsuga* *chinensis* , *Exbucklandia* *tonkinensis* and *Disanthus* *cercidifolia* subsp. *longipes* .

The seed plant flora of Mount Jinggangshan region includes many endemics genera and species. There are 44 genera endemics to China and 1146 species endemics to China (in 424 genera and 131 families), accounting for 17.53% and 39.52% of all in China respectively. The number of endemics genera are equivalent to other mounts in rich biodiversity, such as Mount Shennongjia (43 endemic genera), Mount Wuyishan region (38), Wuling Range (37) and Mount Emeishan (39). The important families with more endemic species include Rosaceae, Lauraceae, Theaceae, Labiatae, Papilionaceae, Fagaceae and Ericaceae.

There are 17 local endemic species in Mount Jinggangshan region, a number similar to Mount of Wuyishan region (20 species), such as *Rhododendron* *jinggangshanicum* , *Rhododendron* *strigosum* , *Rhododendron* *xiaoxidongense* , *Neillia* *jinggngahsnensis* , *Rubus* *glandulosocarpus* , *Actinidia* *chinensis* var. *jinggangshanensis* , *Acer* *cordatum* var. *jinggangshanensis* , *Vitis* *jinggangensis* , *Trichosanthes* *jinggangshanica* , *Impatiens* *jinggangensis* , *Impatiens* *jinggangensis* var. *pauciflora* , *Hemiboea* *subacaulis* var. *jiangxiensis* , which are restricted to the Mount Jinggangshan region. *Rhododendron* *kiangsiense* , *Rhododendron* *hypoblematosum* , *Rhododendron* *crassistylum* , *Elaeagnus* *jiangxiensis* and *Gelidocalamus* *stellatus* are centered in the Mount Jinggangshan region, but also occur in the Luoxiao Mountains.

The greater part of the Chinese flora was usually considered to belong to the Holarctic Kingdom, with a small part in the south belonging to the Paleotropic kingdom in the early days [39]. Hungta Chang [40] and Zhengyi Wu [41] successively proposed that the Chinese flora should be a separate floristic kingdom, the East Asian Kingdom, based on its primitiveness, origin and integrity. Based on Wu’s concept, the flora of Mount Jinggangshan region belongs to the East Asian Kingdom, the Sino-Japanese subkingdom, east China Region and south Jiangxi-east Hunan sub-region. As discussed above, the flora of Mount Jinggangshan region is closely related to the flora of Mount Wuyishan region. They share endemic genera and other elements, therefore both of them should be typical of the flora of eastern China.

However, the flora of Mount Jinggangshan region has obvious transitional properties. Many endemic genera are characteristic of either eastern or central China. The Mount Jinggangshan region is also the southeastern boundary for some typical genera of central China. Furthermore, the Mount Jinggangshan region is a corridor between northern and southern China. Renlin Liu implied that the Mount Jinggangshan region has many tropical elements, especially characterized by Fagaceae, Lauraceae, Theaceae, Hamamelidaceae, and Magnoliaceae [42]. These families make up the dominant elements of the evergreen broadleaved forests as in Nanling Mountains.

In conclusion, Mount Jinggangshan region is one of the richest regions in biodiversity in southeastern China and an important refugium for Tertiary relicts. The region in the central section of the Luoxiao Mountains is an important north-south floristic passageway and is also a boundary between the floras of eastern, central and south China.
